# Associations between dietary advice on modified fibre and lactose intakes and nutrient intakes in men with prostate cancer undergoing radiotherapy

**DOI:** 10.48101/ujms.v127.8261

**Published:** 2022-06-10

**Authors:** Lisa Söderström, Marina Forslund, Birgitta Johansson, Anna Ottenblad, Andreas Rosenblad

**Affiliations:** a Region Vastmanland – Uppsala University, Centre for Clinical Research, Vastmanland Hospital Vasteras, Västerås, Sweden; b Department of Food Studies, Nutrition and Dietetics, Uppsala University, Uppsala, Sweden; c Department of Immunology, Genetics, and Pathology, Uppsala University, Uppsala, Sweden; d Medical Affairs, Nutricia Nordica AB, Solna, Sweden; e Department of Medical Sciences, Clinical Diabetology and Metabolism, Uppsala University, Uppsala, Sweden; f Department of Statistics, Stockholm University, Stockholm, Sweden; g Regional Cancer Centre Stockholm-Gotland, Stockholm, Sweden

**Keywords:** Fibre, lactose, nutrient intake, prostate cancer, radiotherapy

## Abstract

**Objectives:**

A variety of non-evidence-based dietary advice on modified fibre and lactose intakes are provided to patients undergoing pelvic radiotherapy to counteract treatment-related bowel symptoms. More knowledge on the nutritional consequences of such advice is needed. This study aimed to explore how advice on modified fibre and lactose intakes during pelvic radiotherapy was associated with nutrient intakes amongst patients with prostate cancer.

**Methods:**

A total of 77 Swedish men who underwent radiotherapy (50/2 Gy + boost 20–30 Gy) in 2009–2014 due to prostate cancer were given dietary advice at radiotherapy onset (baseline) and at 4 and 8 weeks after radiotherapy onset, to modify their fibre and lactose intakes. At baseline, the participants completed a food frequency questionnaire (FFQ) and a 24-h dietary recall. At 4 and 8 weeks, the participants completed the FFQ and a 4-day estimated food record.

Fibre and lactose intakes were measured by intake scores calculated from the FFQs. Multiple linear regression models were used to analyse associations between intake scores and fibre- and lactose-related nutrients.

**Results:**

In adjusted analyses, there were few significant associations between dietary advice on modified fibre and lactose intakes and observed intakes of fibre- and lactose-related nutrients. A more modified lactose intake was thus associated with a lower intake of calcium (*P* = 0.041), whilst a more modified fibre intake was associated with a higher value for the change in intake of vitamin C (*P* = 0.016).

**Conclusions:**

Dietary advice on modified fibre and lactose intake was in most cases not significantly associated with altered nutrient intakes, rather the energy and nutrient intakes were mostly stable during the pelvic radiotherapy. More research is needed on the nutritional consequences of dietary advice on modified fibre and lactose intakes to reach consensus on if they should continue to be provided in the clinic.

## Introduction

Nutrition interventions (NIs) aiming at modifying fibre and lactose intakes, such as fibre supplementation, lactose restriction, reduced intake of insoluble fibres, low-fibre diet or high-fibre diet, have been evaluated in studies aiming to reduce acute and late bowel symptoms caused by pelvic radiotherapy (1–8). Improvements in bowel symptoms, such as reduced incidence and severity of diarrhoea, have also been reported (2, 6, 7, 9). Even though such modifications of the diet have shown some benefits, there is not enough evidence to recommend them as standard care in this patient group (10–12). Nevertheless, a variety of non-evidence-based dietary advice on modified fibre and lactose intakes are provided to patients with prostate or gynaecological cancer before, during and after radiotherapy (13).

A modified fibre intake during radiotherapy may lead to lower intakes of fibres, wholegrains, and fruits and vegetables, components of a healthy diet, which are associated with lower risks of chronic diseases (14). Furthermore, a reduced intake of lactose-containing dairy products may lead to a reduced intake of calcium and vitamin D. Androgen deprivation therapy, which is associated with decreased bone health, is often added to the radiotherapy treatment for prostate cancer. A sufficient intake of calcium and vitamin D is thus important in this group of patients (15).

Our research group has previously evaluated the effects of an NI aimed at modifying fibre and lactose intakes in randomised controlled trials (RCTs) amongst patients with prostate cancer undergoing radiotherapy (5, 7, 8). The NI resulted in a reduced intake of non-recommended foods and an increase of recommended foods in the intervention group, but not in the control group (5). The NI was significantly associated with less flatulence, less blood in stools, an increased loss of appetite and more bloated abdomen. This present study evaluated the nutritional consequences of dietary advice on modified fibre and lactose intakes, knowledge that may contribute to consensus regarding the standard care for this patient group. The primary aim of this present study was to explore how dietary advice on modified fibre and lactose intakes during pelvic radiotherapy was associated with nutrient intakes in patients with prostate cancer. The hypothesis was that modified fibre and lactose intakes were associated with intakes of fibre- and lactose-related nutrients. The secondary aim was to describe how dietary advice on modified fibre and lactose intakes influenced daily intakes of related food categories.

## Methods

### Study design and setting

This prospective study applied secondary analyses on data collected within an RCT described in detail elsewhere (5). All data were collected within the RCT. The 26-month multi-centre RCT, conducted at three Swedish hospitals between 2009 and 2014, evaluated the effects of modified fibre and lactose intakes on radiotherapy-induced bowel symptoms and health-related quality of life.

### Participants

**Figure 1:**
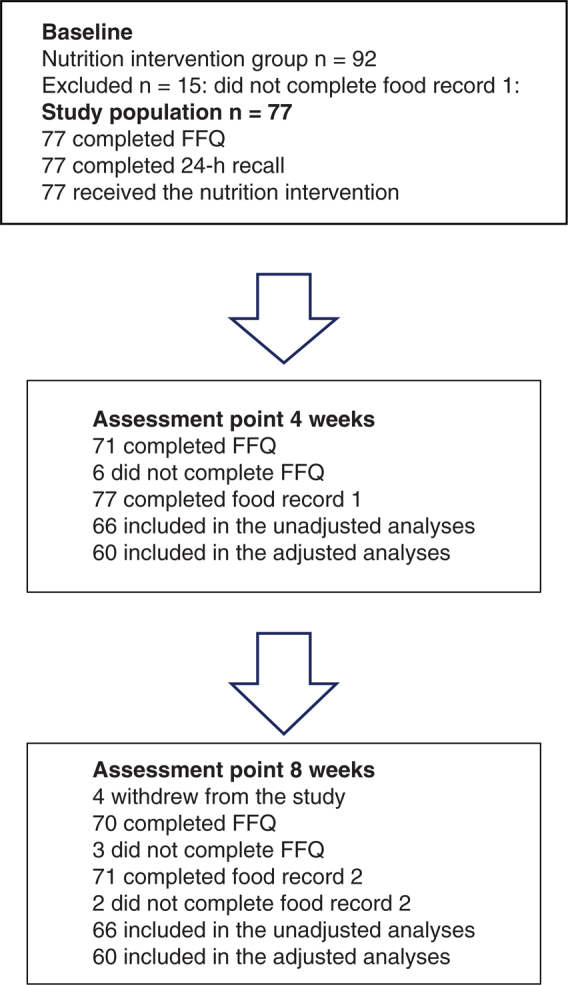
Flow chart. Note: baseline, start of radiotherapy. FFQ: food frequency questionnaire.

Of the 92 patients with prostate cancer randomised to the NI group in the RCT (dietary advice described below), 77 (83.7%) patients fulfilled the inclusion criterium of having a completed 4-day estimated food record at 4 weeks after the start of radiotherapy and were, thus, included in this present study (Figure 1). The participants received curative radiotherapy to the prostate, seminal vesicles and pelvic lymph nodes in 2 Gy fractions up to 50 Gy, and a boost to the prostate (intensity-modulated radiation therapy (IMRT)/volumetric arc therapy (VMAT), brachytherapy, proton or photon) up to a total of 70–80 Gy, during 7–8 weeks, depending on which clinic they were treated at (Table 1) (5). Endocrine treatment was given to most participants (74%).

**Table 1:** Demographic information on the study population: patients with prostate cancer undergoing pelvic radiotherapy, who received a nutrition intervention in a randomised controlled trial.

Variable	Value
Age (years), mean (SD), median (IQR)	66.9 (5.4), 66.0 (63.0, 72.0)
–≤70 years, *n* (%)	52 (67.5)
–>70 years, *n* (%)	25 (32.5)
Height (cm), mean (SD), median (IQR)	178.0 (6.2), 178.0 (174.0, 183.0)
Weight (kg), mean (SD), median (IQR)	87.7 (13.7), 87.1 (75.7, 96.2)
BMI (kg/m^2^), mean (SD), median (IQR)	
–Baseline	27.6 (3.6), 27.5 (25.0, 30.1)
–4 weeks	27.6 (3.6), 27.6 (25.0, 29.9)
–8 weeks	27.6 (3.6), 27.8 (25.3, 29.6)
Scored PG-SGA total score, mean (SD), median (IQR)	2.4 (1.9), 2.0 (2.0, 3.0)
Scored PG-SGA global rating, *n* (%)	
A: well-nourished	74 (96)
B: at risk/moderately malnourished	3 (4)
Marital status, *n* (%)	
–Married/cohabiting	65 (84)
–Single/divorcee	10 (13)
–Unknown	2 (3)
Smoking, *n* (%)	
–Current smoker	7 (9)
–Never smoked	30 (39)
–Former smoker	36 (47)
–Unknown	4 (5)
Diabetes, *n* (%)	
–Yes	9 (12)
–No	68 (88)
Treatment modality, *n* (%)	
–IMRT/VMAT + IMRT/VMAT boost	32 (42)
–IMRT/VMAT + brachytherapy boost	24 (31)
–IMRT/VMAT + proton or photon boost	21 (27)
Endocrine treatment, *n* (%)	
–Yes	74 (96)
–No	3 (4)

BMI: body mass index; IMRT: intensity-modulated radiation therapy; IQR: interquartile range; PG-SGA: the scored Patient-Generated Subjective Global Assessment tool; VMAT: volumetric arc therapy.

### Nutrition Intervention

The participants were at radiotherapy onset, mid-treatment (4 weeks) and end of treatment (8 weeks) advised by research dietitians to modify their fibre intake by reducing their intake of insoluble fibres and increasing their intake of soluble fibres, and to reduce their lactose intake, during the entire study period of 26 months. The dietary advice was presented as foods recommended and foods not recommended. Dietary advice and recommendations were given on replacing wholegrain products: seeds or bran with bread baked with wheat, oat or light rye flour; cereals from maize; wheat or oats; and regular white rice and pasta. Fruits and vegetables with tough seeds or skins were replaced with peeled, canned or tender fruits and cooked or tender vegetables. Leguminous plants and nuts were not recommended. High-lactose dairy products were replaced with lactose-reduced or lactose-free dairy products fortified with calcium and vitamin D. No study-specific targets regarding intakes in grams or percentages were defined within the NI.

### Assessment at baseline and at 4 weeks (mid-treatment) and 8 weeks (end of radiotherapy)

The baseline assessment was completed at the start of radiotherapy, before randomisation. Background data were obtained from medical records and from the participants during the baseline assessment (Table 1). Weight and height were collected from the medical records or were self-reported at baseline, and self-reported weight was collected at follow-ups. At baseline, the participants completed a study-specific food frequency questionnaire (FFQ) to assess the intake of 61 fibre- or lactose-containing food items the preceding month. The participants also completed a 24-h dietary recall at baseline, that is, a structured interview to capture the pre-intervention energy and nutrient intake, in which all food and beverage intakes during the preceding day were recorded. At 4 weeks (mid-treatment) and 8 weeks (end of radiotherapy), the participants completed the FFQ and a 4-day estimated food record (Supplementary Table 1).

### Food frequency questionnaire

A study-specific FFQ was used to assess the intake of 61 fibre- or lactose-containing food items the preceding month (Supplementary Table 2). Answers were given on an eight-level ordinal scale ranging from ‘never/less than once a month’ to ‘≥3 times/day’; 4-day estimated food records were used at mid-treatment and end of treatment to assess energy and nutrient intakes. All foods and beverages were prospectively recorded (type of food, brand name and portion size) during 4 consecutive days, including a weekend, to capture differences in food intake between weekdays and weekends. The amount of food and drink could be recorded using household measures or estimated using a booklet developed by the Swedish Food Agency, containing photographs of portion sizes (16).

### Fibre and lactose intakes

Fibre and lactose intake scores (FIS and LIS) were calculated from FFQ data. Only food items consumed ≥2 times/month were used for these calculations. The remaining 30 items were categorised into the following six categories:

1. Fibre categories:
  - recommended grain products (*n* = 8)
  - not recommended grain products (*n* = 3)
  - recommended vegetables (*n* = 6)
  - not recommended vegetables (*n* = 6)
2. Dairy categories:
  e. dairy products with low lactose content (*n* = 3)
  f. dairy products with high lactose content (*n* = 4).

For each category, the number of intakes/day was calculated, and the categories were then dichotomised using the median number of intakes/day in each category as cut-off, with intakes ≤ median for categories a, c and e scored 0 and otherwise scored 1, and intakes ≥ median for categories b, d and f scored 0 and otherwise scored 1. An additional question regarding the use of lactose-reduced dairy products was scored 1 (‘yes’) or 0 (‘no’). Thus, each participant received an FIS ranging from 0 to 4, and an LIS ranging from 0 to 3, for the assessment points at mid-treatment and end of treatment. Higher FIS and LIS scores, thus, corresponded to more modified intakes.

### Ethical considerations

The RCT was approved by the Uppsala Regional Ethical Review Board (Dnr. 2009/209). All patients provided their written informed consent to participate in the study.

### Statistical analyses

The software DIETIST XP for Windows, version 3.2 (Kost och näringsdata AB, Stockholm, Sweden), based on the Swedish Food Composition database (version 2013-10-04), was used to obtain energy and nutrient intakes from the 24-h recall and the 4-day estimated food records. The average daily intakes on the 4 recorded days in the 4-day estimated food records were used in the analyses. If more than 4 days had been recorded (*n* = 5), including a weekend, 2 weekdays and 2 weekend days were used in the calculations. If only weekdays had been recorded, the first 4 days were used for calculations. Results from the 24-h recall and the 4-day estimated food records are presented alongside intakes amongst men in similar age from a national dietary survey (hereinafter referred to as the reference group) (17), as well as the Nordic Nutrition Recommendations (NNR) 2012 for the given age group (Table 2) (14).

**Table 2:** Energy and nutrient intakes, presented as means (SD) and as the 5th and 95th percentile, obtained from the 24-h recall at baseline, and the 4-day estimated food records 1 and 2, completed 4 and 8 weeks after baseline.

	24 h recall Baseline	4-day food record Mid-treatment	4-day food record End of treatment	Riksmaten Adults 2010–11	NNR 2012
	*n* = 77	*n* = 77	*n* = 71	Men 65–80 years	Men ≥ 61 years
kJ Mean (SD) 5th percentile 95th percentile	7909 (2373) 3866 11209	8516 (2029) 5177 11986	8834 (1731) 5645 11317	8715 (2301) 5213 12970	N/A
kcal Mean (SD) 5th percentile 95th percentile	1890 (567) 926 2682	2035 (485) 1236 2864	2110 (414) 1349 2704	2083 (550) 1246 3100	N/A
Protein, g Mean (SD) 5th percentile 95th percentile	78.5 (22.2) 41 117	84.9 (20.7) 50 124	87.3 (19.0) 53 120	83.8 (21.7) 52 121	N/A
Fat, g Mean (SD) 5th percentile 95th percentile	76.6 (30.9) 35 139	84.0 (24.5) 42 130	87.3 (21.4) 55 123	79.8 (27.9) 41 126	N/A
Carbohydrates, g Mean (SD) 5th percentile 95th percentile	203 (76) 83 310	209 (65) 99 312	221 (57) 115 308	223 (68) 113 333	N/A
Fibre, g Mean (SD) 5th percentile 95th percentile	18.8 (7.9) 8.5 33.7	16.4 (5.8) 7.2 26.9	17.0 (5.6) 6.9 27.3	22.5 (7.6) 11.3 35.8	≥35
Calcium, mg mean (SD) 5th percentile 95th percentile	875 (433) 306 1800	830 (267) 409 1402	885 (303) 441 1583	885 (312) 398 1440	800
Potassium, mg Mean (SD) 5th percentile 95th percentile	2926 (918) 1430 4343	3048 (776) 1772 4308	3173 (743) 1841 4339	3392 (904) 2007 4962	3500
Magnesium, mg Mean (SD) 5th percentile 95th percentile	283 (87) 137 458	291 (69) 175 422	302 (61) 182 406	347 (97) 198 511	350
Iron, mg Mean (SD) 5th percentile 95th percentile	9.8 (5.8) 2.9 19.4	10.2 (3.5) 5.3 18.1	9.9 (2.9) 4.3 14.4	11.0 (3.7) 5.4 17.0	9
Zinc, mg Mean (SD) 5th percentile 95th percentile	10.7 (3.4) 5.6 16.6	10.7 (3.1) 5.8 16.8	11.2 (2.8) 6.5 15.9	10.9 (3.1) 6.0 16.8	9
Selenium, μg Mean (SD) 5th percentile 95th percentile	40 (20) 13 81	48 (17) 24 87	51 (17) 26 81	50 (19) 26 84	60
Vitamin D, μg Mean (SD) 5th percentile 95th percentile	7.1 (7.5) 1.6 23.2	8.0 (4.2) 2.5 14.1	9.5 (6.0) 2.8 19.8	9.1 (5.9) 3.2 19.5	10–20^a^
Vitamin B6, mg Mean (SD) 5th percentile 95th percentile	1.9 (0.8) 0.7 3.4	2.0 (0.7) 1.0 3.4	2.1 (0.6) 1.1 3.4	2.2 (0.9) 1.2 4.5	1.5
Folate, μg Mean (SD) 5th percentile 95th percentile	238 (86) 118 402	266 (187) 128 414	278 (189) 141 380	279 (104) 158 443	300
Vitamin C, mg Mean (SD) 5th percentile 95th percentile	88 (67) 14 227	90 (60) 24 234	110 (71) 25 265	110 (57) 37 211	75

Data from the nationwide dietary survey Riksmaten adults 2010–2011 and recommended intakes (RI) for NNR 2012 are included as reference values.

Note: μg: microgram; mg: milligram; NA: not applicable; NNR: Nordic Nutrition Recommendations; SD: standard deviation.

a People 75 years or older, n = 5 in the study population, are recommended 20 μg per day.

To describe how dietary advice on modified fibre and lactose intakes influenced daily intakes of related food categories, the food items in the study-specific FFQ were used.

Only food items that were classified as wholegrain products according to the Swedish Food Agency were categorised as wholegrains (18) (*n* =15). Thus, the FFQ, which originally contained 61 items, was reduced to 44 items and was categorised as wholegrain products (*n* = 15), fruits and vegetables (*n* = 14) or dairy products (*n* = 15) (Supplementary Table 3). The number of daily intakes was calculated as the proportion of participants reporting consumption in the respective categories: 0 times/day, 1–2 times/day, 3–4 times/day or ≥5 times/day at baseline, 4 weeks and 8 weeks.

Categorical data are presented as frequencies and percentages, *n* (%), whilst continuous data are given as means with accompanying standard deviations (SDs), supplemented with medians and interquartile ranges (IQRs).

Separate multiple linear regression models were used to analyse associations between FIS (independent variable) and kJ, potassium (mg), magnesium (mg), iron (mg), zinc (mg), selenium (μg), vitamin B6 (μg), folate (μg) and vitamin C (mg) (dependent variables), as well as between LIS (independent variables) and kJ, calcium (mg) and vitamin D (μg) (dependent variables), at mid-treatment and end of treatment. All models were adjusted for age (years), body mass index (BMI; kg/m^2^), marital status, Patient-Generated Subjective Global Assessment (PG-SGA) total score (9, 19), smoking habits, diabetes and energy and nutrient intakes at baseline. All statistical analyses were performed in IBM SPSS Statistics, with two-sided *P*-values < 0.05 considered statistically significant.

## Results

### Participant characteristics

The participants’ mean (SD) age was 66.9 (5.4) years; the majority were married or cohabiting (*n* = 65; 84%), well-nourished according to PG-SGA (*n* = 74; 96%) and non-smokers (*n* = 66; 86%) (Table 1). All participants had completed the 24-h dietary recall at baseline, as well as the mid-treatment 4-day estimated food record. Most participants (*n* = 71; 92%) had completed the 4-day estimated food record at the end of treatment. Four participants withdrew from the study (Figure 1).

### Pre-intervention nutrient intakes

Estimated pre-intervention nutrient intakes were lower than intakes in the reference group (17). The participants met the recommended intake (RI) according to NNR 2012 for calcium, iron, zinc, vitamin B6 and vitamin C. However, fibre, potassium, magnesium, selenium, vitamin D and folate intakes were lower than recommended (Table 2).

**Figure 2:**
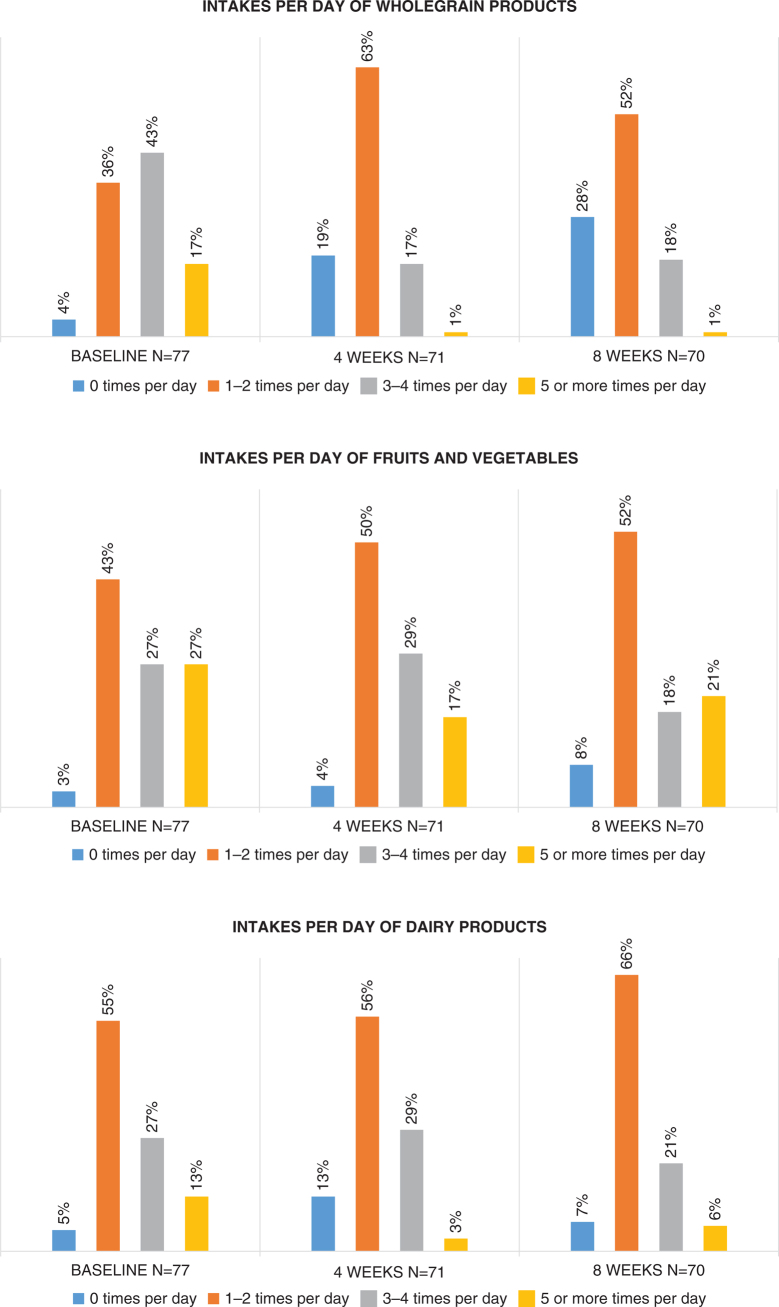
Proportions of participants reporting given numbers of intakes per day in the food categories wholegrains, fruits and vegetables, and dairy products, at baseline, mid-treatment and end of treatment. Descriptive data calculated from food frequency questionnaires completed within the randomised controlled trial.

The 44 food items from the FFQ were sorted into three food categories: wholegrain products, fruits and vegetables or dairy products. During the month preceding baseline, 60% had consumed wholegrain products ≥3 times/day, 54% reported ≥3 intakes/day of fruits and vegetables and 40% had consumed dairy products ≥3 times/day (Figure 2).

### Energy and nutrient intakes at follow-up

Overall, the estimated energy and nutrient intakes remained stable or increased from baseline to 8 weeks (Table 2). Fibre intake decreased from baseline to 4 weeks and then remained stable at the lower level from 4 to 8 weeks. Overall, the participants’ intakes at the end of treatment were higher than or similar to intakes in the reference group, except for fibre, potassium, magnesium and iron. Despite improved nutrient intakes at the end of treatment, the participants did not meet the RI of NNR 2012 for potassium, magnesium, selenium, vitamin D or folate.

In the adjusted analyses, participants who had lowered their intake of lactose products as advised (i.e. higher LIS score) had lower calcium intake at 8 weeks (*P* = 0.041). Participants with a larger change in fibre intake between 4 and 8 weeks (i.e. relatively higher FIS score at 8 weeks compared to 4 weeks) had not only a larger increase in vitamin C intake (*P* = 0.009) but also a larger decrease in selenium intake (*P* = 0.039) in the unadjusted analyses (Table 3). The association between a larger change in fibre intake between 4 and 8 weeks and increased vitamin C intake was statistically significant also in the adjusted analyses (*P* = 0.016).

**Table 3:** Results from the unadjusted and adjusted multiple linear regression analyses of data from 4 to 8 weeks, and change from 4 to 8 weeks, analysing associations between modified fibre and lactose intakes and nutrient intakes.

Models	Unadjusted analyses (*n* = 66)			Adjusted analyses^a^ (*n* = 60)		
	Slope coefficient	95% CI	*P*-value^b^	Slope coefficient	95% CI	*P*-value^b^
**4 weeks**						
kJ FIS LIS	11.0 383.1	−406.5, 428.4 −345.0, 1111.1	0.958 0.297	−118.3 156.6	−537.4, 300.8 −517.6, 830.8	0.573 0.643
Calcium, mg LIS	−75.1	−175.5, 25.3	0.140	−59.9	−150.7, 31.0	0.192
Potassium, mg FIS	−9.8	−164.7, 145.1	0.899	−103.4	−273.6, 66.9	0.229
Magnesium, mg FIS	1.0	−12.9, 14.8	0.891	−7.2	−20.2, 5.8	0.271
Iron, mg FIS	0.3	−0.4, 1.1	0.371	0.2	−0.8, 1.1	0.710
Zinc, mg FIS	−0.2	−0.9, 0.4	0.475	−0.5	−1.2, 0.2	0.152
Selenium, μg FIS	−0.4	−4.0, 3.2	0.837	1.0	−3.3, 5.2	0.646
Vitamin D, μg LIS	0.5	−1.0, 2.1	0.493	0.6	−1.1, 2.2	0.509
Vitamin B6, μg FIS	0.0	−0.1, 0.2	0.507	0.0	−0.1, 0.2	0.626
Folate, μg FIS	30.9	−9.6, 71.4	0.133	34.8	−14.3, 83.9	0.161
Vitamin C, mg FIS	5.1	−6.9, 17.1	0.396	6.9	−5.9, 19.7	0.286
**8 weeks**						
kJ FIS LIS	81.6 −108.8	−325.7, 488.9 −796.3, 578.7	0.690 0.753	−154.6 −225.1	−523.4, 214.1 −819.1, 368.9	0.404 0.450
Calcium, mg LIS	−91.8	−203.3, 19.6	0.105	−125.2	−245.3, −5.1	0.041
Potassium, mg FIS	−21.5	−192.9, 149.9	0.803	−60.4	−217.4, 96.6	0.444
Magnesium, mg FIS	−4.0	−18.0, 10.1	0.576	−9.3	−22.1, 3.6	0.153
Iron, mg FIS	0.2	−0.5, 0.9	0.533	0.1	−0.7, 0.8	0.866
Zinc, mg FIS	−0,2	−0.8, 0.5	0.555	−0.3	−1.0, 0.4	0.402
Selenium, μg FIS	−2.1	−6.0, 1.9	0.298	−3.3	−7.7, 1.1	0.141
Vitamin D, μg LIS	0.3	−2.0, 2.7	0.776	0.5	−2.3, 3.3	0.695
Vitamin B6, μg FIS	0.0	−0.1, 0.2	0.637	0.0	−0.1, 0.2	0.851
Folate, μg FIS	−14.0	−59.0, 31.0	0.536	−22.3	−75.6, 30.9	0.404
Vitamin C, mg FIS	13.3	−2.7, 29.3	0.101	6.9	−8.9, 22.8	0.384
**Change from 4 to 8 weeks^c^**						
kJ FIS LIS	−152.5 −24.8	−549.0, 244.0 −630.2, 580.7	0.445 0.935	−59.8 99.5	−533.9, 414.2 −589.6, 788.6	0.801 0.773
Calcium, mg LIS	−79.4	−172.4, 13.6	0.093	−84.6	−192.2, 23.0	0.121
Potassium, mg FIS	−104.6	−267.9, 58.8	0.206	−96.1	−276.8, 84.6	0.291
Magnesium, mg FIS	−6.6	−21.0, 7.8	0.361	−7.7	−23.8, 8.4	0.343
Iron, mg FIS	0.4	−0.4, 1.1	0.362	0.4	−0.5, 1.4	0.380
Zinc, mg FIS	−0.5	−1.1, 0.2	0.137	−0.7	−1.5, 0.0	0.063
Selenium, μg FIS	−3.9	−7.5, −0.2	0.039	−2.8	−7.1, 1.5	0.196
Vitamin D, μg LIS	0.9	−1.2, 2.9	0.398	1.1	−1.2, 3.4	0.336
Vitamin B6, μg FIS	−0.0	−0.1, 0.1	0.864	0.0	−0.1, 0.1	0.990
Folate, μg FIS	−4.4	−61.6, 52.8	0.877	1.3	−65.9, 68.4	0.970
Vitamin C, mg FIS	15.8	4.0, 27.5	0.009	17.2	3.3, 31.1	0.016

Nutrient intake is used as the dependent variable, and fibre intake score (FIS) and lactose intake score (LIS) as independent variables in all models.

Note: Confidence intervals (CIs) are presented as lower bound and upper bound.

a All models adjusted for age, BMI, Patient-Generated Subjective Global Assessment total score, marital status, smoking habits, diabetes (using living alone, never smoked and no diabetes as reference) and the specific energy and nutrient intakes at baseline.

b P-values are considered statistically significant if P < 0.05.

c Change from 4 to 8 weeks was calculated as 8 weeks to 4 weeks for nutrients, the FIS and the LIS, respectively.

The proportion of participants reporting the lowest number of intakes per days of wholegrain products (≤2 times/day) had increased from baseline to end of treatment, whilst the proportion who reported eating wholegrains ≥3 times/day decreased (Figure 2). The proportion of participants with less frequent intake of fruits and vegetables (≤2 times/day) increased after receiving the dietary advice, and the proportion of participants reporting eating fruits and vegetables ≥3 times/day at follow-up at 4 and 8 weeks had decreased, compared with baseline. Also, a larger proportion of participants reported consuming dairy products ≤2 times/day at follow-up at 4 and 8 weeks, whilst the proportion reporting more frequent intakes decreased, compared with baseline.

## Discussion

Dietary advice on modified fibre and lactose intakes was in most cases not significantly associated with altered nutrient intakes, rather the energy- and nutrient intakes were mostly stable during the pelvic radiotherapy. However, an important finding was the association between a more modified lactose intake and a lower calcium intake.

A sufficient calcium intake is important for patients with prostate cancer who are undergoing androgen deprivation therapy, due to its association with impaired bone health (15). A weak negative correlation has been observed between degree of lactose intolerance and calcium intake in patients with prostate cancer, suggesting that lactose intolerance may affect the quantity of calcium in the diet (20). Lactase deficiency does not seem to affect calcium absorption in adults, but if dairy products are avoided or reduced as a consequence of lactose intolerance, this could predispose individuals to reduced bone health (21).

The participants in the present study were advised to choose lactose-reduced dairy products fortified with calcium and vitamin D, the mean intake of calcium increased after the dietary counselling at mid-treatment, and, overall, the participants met the NNR 2012 recommendations for calcium intake throughout the study period. The association between a more modified lactose intake and lower calcium intake may be explained by some of the participants choosing to reduce their frequency of consumption of dairy products as a consequence of the dietary advice, as reflected in the descriptive FFQ data. Here, the role of the dietitian is the key: given their expertise on how to individualise dietary advice to patients’ habitual diets, preferences and prior knowledge, dietitians can guide patients to choose fortified alternatives in order to secure an adequate nutrient intake.

However, in a study from the UK including patients who had completed radiotherapy for pelvic cancer, only a minority (16%) consulted dietitians about their symptoms. Still, half of the patients had made dietary changes to manage gastrointestinal symptoms, such as eliminating dairy products and fruits from their diet (22). Making such dietary changes without consulting a dietitian could increase the risk of an imbalanced diet if foods are excessively excluded.

The association between a more modified fibre intake and a higher value for the change in the intake of vitamin C from 4 to 8 weeks indicates that the participants did not drastically reduce their consumption of fibres, and that they were successful in replacing fruits and vegetables with tough seeds or skins with peeled, canned or tender fruits and cooked or tender vegetables. This assumption is supported by the fact that >90% of the participants reported eating fruits and vegetables at least once a day at follow-up (Figure 2), and by the increasing mean intake of vitamin C from baseline throughout the study period, again highlighting the benefits of counselling from a dietitian when patients are advised to modify their fibre intakes during radiotherapy.

Similar levels of intakes and patterns regarding energy and nutrients were observed in a previous RCT from our research group (5), where patients with prostate cancer undergoing radiotherapy to the prostate gland received the same dietary advice and completed 4-day estimated food records in the same way as in this present study. The similar patterns with increasing intakes in the two studies indicate possible benefits of receiving dietary advice from a dietitian when patients are to make changes to their diets during radiotherapy. In another study assessing the impact of an anti-fermentative diet on dietary intake and body composition in a group of patients with prostate cancer undergoing radiotherapy (23), all energy and nutrient intakes decreased when following the anti-fermentative diet and did not recover to baseline values during the study period. The authors highlighted that nutritional support is essential for maintenance of nutritional status when patients with prostate cancer are provided with dietary restrictions. More sessions with the dietitian for patients in need of more support were emphasised in a previous study by our research group, exploring the experiences of the NI aiming at modifying fibre and lactose intakes in an RCT amongst patients with prostate cancer undergoing radiotherapy (24).

Another RCT concluded that a higher fibre intake could be beneficial during pelvic radiotherapy due to positive effects on gastrointestinal symptoms (6). In that study, energy intake at baseline was similar to our study, and fibre intake in the high-fibre group at the final week of radiotherapy was similar to fibre intake at the end of treatment in the present study. A higher fibre intake enhances the production of short chain fatty acids arising from fermentation of mainly soluble fibres by gut bacteria, which may be beneficial to gut health and have anti-inflammatory effects (25, 26).

Despite the positive trend with increasing intakes of nutrients from baseline to 8 weeks, the participants in the present study did not meet the RI of NNR 2012 for potassium, magnesium, selenium, vitamin D or folate. Wholegrains, vegetables, legumes and dairy products are sources of these nutrients, and these are foods that the participants were recommended to avoid, modify or substitute. Thus, it cannot be ruled out that modified fibre and lactose intakes may have affected the intakes of these nutrients. These findings indicate the relevance of dietitians closely monitoring micronutrient intake and ensuring adequate supplementation when needed.

### Strengths and limitations of this study

A strength of the present study is the exploration of associations between modified fibre and lactose intakes and nutrient intakes in this patient group, an area rarely explored in previous research. A weakness is the small sample size limiting the power of the statistical analyses. Furthermore, self-reported dietary assessments, such as 24-h recall, FFQ and 4-day dietary food records are associated with measurement errors, such as recall bias (27), and the estimates of energy and nutrient intakes from these are not entirely comparable. To diminish measurements errors for the food records, a booklet with photographs (16) intended to facilitate the estimations of portion sizes, and verbal and written instructions from the dietitians on how to complete the 4-day estimated food records and the FFQ were provided. A limitation regarding the dietary advice is that no goals were formulated in terms of percentages, grams or number of portions, making it difficult to evaluate exactly how much the participants modified their intakes. The FIS and LIS scores were constructed from the FFQ, which did not include portion sizes, meaning that intakes could be very small or quite large; this needs to be taken into consideration when interpreting the results. In addition, the FFQ used in the present study was study-specific, and its validity has not yet been evaluated. Finally, the comparisons between the pre-intervention intakes at baseline from the 24-h recall and intakes in the reference group should be interpreted with some caution. The 24-h recall may not have fully captured usual intakes amongst the participants due to within-subject variation in food intake, and the small sample size was used (27). Another factor that might have contributed to lower intakes amongst the participants at baseline is that the 24-h recall was conducted during the first days of radiotherapy, and it is not unlikely that the treatment had a negative effect on the participants’ usual dietary habits and appetite. This was an observational study; hence, we cannot draw clear conclusions that modified fibre and lactose intakes increased or decreased nutrient intakes; we can only state that there were few statistically significant associations. To draw any conclusions on cause and effect, RCTs with adequate power must be conducted.

## Conclusions

Dietary advice on modified fibre and lactose intakes was in most cases not significantly associated with altered nutrient intakes in patients with prostate cancer undergoing pelvic radiotherapy. Rather, the energy- and nutrient intakes were mostly stable during the pelvic radiotherapy. A more modified intake of lactose was, however, significantly associated with a lower intake of calcium, and if patients are advised to reduce their lactose intake during pelvic radiotherapy, they should, thus, receive appropriate nutritional support. A more modified fibre intake was associated with an increased vitamin C intake. More research is needed on the nutritional consequences of dietary advice on modified fibre and lactose intakes to reach consensus on if they should continue to be provided in the clinic.

## Disclosure statement

No potential conflicts of interest have been reported by the authors.

## Funding

This work was based on data collected within a RCT, supported by the Swedish Cancer Society, under grant number CAN 2008/799; the Uppsala-Örebro Regional Research Council, under grant number RFR 386491; and the Faculty of Medicine at Uppsala University.

## Notes on contributors

***Lisa Söderström***, is a dietitian and obtained her PhD in medical sciences in 2016 at Uppsala University. Previously, she was a lecturer in nutrition at Uppsala University and is currently working at the Centre for Clinical Research in Västerås, Sweden.

***Marina Forslund***, is a registered dietitian and obtained her PhD in medical sciences in 2020 at Uppsala University. She is currently working as a clinical dietitian in oncology at a hospital in Gävle, Sweden.

***Birgitta Johansson***, is a registered nurse and obtained her PhD in 2000. She is an associate professor in caring sciences and a senior lecturer in oncology nursing at Uppsala University. She has authored more than 60 peer-reviewed articles in international scientific journals.

***Anna Ottenblad***, (previously Pettersson) is a dietitian and obtained her PhD in medical sciences in 2014 at Uppsala University. In addition to clinical roles, she has held positions in management and project management in various healthcare organizations. She is currently working as Medical Affairs Manager at Nutricia Nordica, Sweden.

***Andreas Rosenblad***, previously *Karlsson*, born 1973, obtained his PhD in statistics from Uppsala University in 2006 and has been an associate professor in applied medical statistics and epidemiology at Uppsala University since 2014. Previously, he was a senior lecturer in statistics at Stockholm University. He is currently an affiliated researcher at the Department of Medical Sciences, Uppsala University and the Department of Neurobiology, Care Sciences and Society, Karolinska Institute, and employed as a statistician at the Regional Cancer Centre Stockholm-Gotland. He is an author of more than 70 peer-reviewed articles in international scientific journals.

## ORCID

Lisa Söderström https://orcid.org/0000-0002-8367-1189

Marina Forslund https://orcid.org/0000-0002-5458-078X

Birgitta Johansson https://orcid.org/0000-0001-6226-6849

Anna Ottenblad https://orcid.org/0000-0001-7672-2999

Andreas Rosenblad https://orcid.org/0000-0003-3691-8326

## Supplementary Material

Associations between dietary advice on modified fibre and lactose intakes and nutrient intakes in men with prostate cancer undergoing radiotherapy

Associations between dietary advice on modified fibre and lactose intakes and nutrient intakes in men with prostate cancer undergoing radiotherapy

Associations between dietary advice on modified fibre and lactose intakes and nutrient intakes in men with prostate cancer undergoing radiotherapy
