# Supplementary material for: Associations between dietary advice on modified fibre and lactose intakes and nutrient intakes in men with prostate cancer undergoing radiotherapy
Source: Ups J Med Sci. 2022 Jun 10;127:10.48101/ujms.v127.8261. doi: 10.48101/ujms.v127.8261 (PMC9199583; doi:10.48101/ujms.v127.8261)
Supplement: Associations between dietary advice on modified fibre and lactose intakes and nutrient intakes in men with prostate cancer undergoing radiotherapy [file UJMS-127-8261-s001.pdf]

Table S1. Dietary data collection and methods.

	Baseline, RT onset	4 weeks after RT onset	8 weeks after RT onset
Dietary advice by research dietitian	Face-to-face	Face-to-face	By telephone
24-h dietary recall	Face-to-face		
4-day estimated food records		Self-reported	Self-reported
FFQ	Self-reported	Self-reported[a]	Self-reported[a]

Note: RT, radiotherapy; FFQ, food frequency questionnaire. The baseline appointment with the research dietitian was conducted at the hospital where the patient received their radiotherapy treatment. A total of three registered dietitians, one at each hospital, gave dietary advice. The participants received the nutrition intervention after the baseline assessment was completed. The 4-day estimated food records were handed in to the dietitian prior to each follow-up assessment. [a] Collected by post.
