# Supplementary material for: Associations between dietary advice on modified fibre and lactose intakes and nutrient intakes in men with prostate cancer undergoing radiotherapy
Source: Ups J Med Sci. 2022 Jun 10;127:10.48101/ujms.v127.8261. doi: 10.48101/ujms.v127.8261 (PMC9199583; doi:10.48101/ujms.v127.8261)
Supplement: Associations between dietary advice on modified fibre and lactose intakes and nutrient intakes in men with prostate cancer undergoing radiotherapy [file UJMS-127-8261-s002.pdf]

Study number: Date:

Table S2.

How often do you eat the following foods?

We ask you to state your intake of the specified foods as carefully as you can. Check the box that best matches your intake of each individual food, i.e. ***how often you usually eat it****. The answers should be valid for* ***the last month****.*
(Check the "Never" column if you have not eaten the food in question during the last month.)

	Per day			Per week		Per month		Never
	≥3 times	2 times	1 time	2 times	1 time	2 times	1 time	< 1 time / month
Milk								
Sour milk								
Yoghurt								
Cream								
Crème fraiche products								
Whey cheese and / or whey butter								
Ice cream								
Feta cheese, sheep cheese								
Sour cream								
Quark								
Soft cheese, cream cheese								
Dessert cheese								
Milda Mat (ready-made sauce / cream)								
Kelda (cream / ready-made soups)								
Ready-to-eat rice pudding snack								
Potatoes								
Fresh root vegetables								
Cooked root vegetables								
Root vegetables in dishes								
Raw vegetables								
Cooked vegetables								
Vegetables in dishes								
Beans brown / white / green etc								
Lentils								
Pea soup								
Rice, polished / white								
Brown rice / whole grain rice								
Pasta (macaroni, etc.), noodles								

Study number: Date:

CONTINUE ON NEXT PAGE - PLEASE TURN PAGE!

	Per day			Per week		Per month		Never
	≥3 times	2 times	1 time	2 times	1 time	2 times	1 time	< 1 time / month
Wholegrain pasta								
Berries								
Fresh fruit								
Peeled fresh fruit								
Canned fruit								
Dried fruit								
Nuts								
Almond								
Seeds; sunflower, flax, sesame								
Rolled oats								
Rolled oats with wheat bran								
Rolled rye flakes								
Barleycorn / pearl-barley								
Corn flakes								
Whole grain cereals, bran flakes								
Muesli (base)								
Muesli with fruit								
Wheat germ								
Wheat bran								
Oat bran								
Crispbread, rye								
Crispbread, oat								
Crispbread, wheat								
Soft bread, white								
Soft bread, sifted rye flour								
Soft bread, whole grains								
Soft bread with seeds, kernels								
Wheat bread (buns and cakes)								
Crusts, wheat								
Crusts, rye								
Crusts, graham								
Biscuits, whole grain								
Biscuits, wheat								

1. What type of milk do you use?
Milk 0.1% fat ❑ Milk 0.5 % fat ❑ Milk 1.5% fat ❑ Milk 3% fat ❑ Other / Don't know ❑ Do not use milk ❑

2. Do you use lactose-reduced products (e.g. low-lactose milk or lactose-free milk / sour milk / cream)?
Yes ❑ No ❑

If yes, which?...
