# Supplementary material for: Associations between dietary advice on modified fibre and lactose intakes and nutrient intakes in men with prostate cancer undergoing radiotherapy
Source: Ups J Med Sci. 2022 Jun 10;127:10.48101/ujms.v127.8261. doi: 10.48101/ujms.v127.8261 (PMC9199583; doi:10.48101/ujms.v127.8261)
Supplement: Associations between dietary advice on modified fibre and lactose intakes and nutrient intakes in men with prostate cancer undergoing radiotherapy [file UJMS-127-8261-s003.pdf]

Table S3. Food items from the food frequency questionnaire sorted into the categories wholegrains, fruits and vegetables, and dairy products.

Wholegrains n = 15	Fruits and vegetables n = 14	Dairy products n = 15
Brown rice	Raw roots	Milk
Wholegrain pasta	Boiled roots	Soured milk
Rolled oats	Roots in dishes	Yoghurt
Rolled oats with bran	Raw vegetables	Cream
Rolled rye	Boiled vegetables	Crème fraîche
Barley	Vegetables in dishes	Soft whey butter
Cereals with bran	Beans	Ice cream
Muesli	Lentils	Feta cheese
Muesli with dried fruit	Soup with peas	Sour cream
Rye crisp bread	Berries	Cottage cheese
Oat crisp bread	Fruits with skin	Cream cheese
Whole meal bread	Peeled fruits	Cheese spread
Whole meal bread with seeds	Canned fruits	Ready-to-eat sauce with dairy
Whole meal biscuits	Dried fruits	Ready-to-eat soup with dairy
Whole meal cookies		Rice pudding
